# Torrential tricuspid regurgitation in Ebstein’s anomaly, never seen so clear!

**DOI:** 10.1093/ehjimp/qyae066

**Published:** 2024-07-02

**Authors:** Laura Victoria Torres-Araujo, Jorge Alberto Silva-Estrada, Moisés Jiménez-Santos, Ana María Rosas-Vázquez, Edgar García-Cruz

**Affiliations:** CT Scanner Lomas Altas, Paseo de la Reforma #2608, Lomas Altas, Mexico City, 11950, Mexico; Instituto Nacional de Cardiología ‘Ignacio Chávez’, Juan Badiano 1, Belisario Domínguez - Sección XVI,Tlalpan, Mexico city, 14080, Mexico; CT Scanner Lomas Altas, Paseo de la Reforma #2608, Lomas Altas, Mexico City, 11950, Mexico; Instituto Nacional de Pediatría, Insurgentes Sur #3700, Insurgentes Cuicuilco, Coyoacán, 04530, Mexico City, Mexico; CT Scanner Lomas Altas, Paseo de la Reforma #2608, Lomas Altas, Mexico City, 11950, Mexico; Instituto Nacional de Cardiología ‘Ignacio Chávez’, Juan Badiano 1, Belisario Domínguez - Sección XVI,Tlalpan, Mexico city, 14080, Mexico; CT Scanner Lomas Altas, Paseo de la Reforma #2608, Lomas Altas, Mexico City, 11950, Mexico; Instituto Nacional de Cardiología ‘Ignacio Chávez’, Juan Badiano 1, Belisario Domínguez - Sección XVI,Tlalpan, Mexico city, 14080, Mexico; Instituto Nacional de Cardiología ‘Ignacio Chávez’, Juan Badiano 1, Belisario Domínguez - Sección XVI,Tlalpan, Mexico city, 14080, Mexico

**Keywords:** 4D flow, cardiac magnetic resonance, congenital heart disease

A 29-year-old male with Ebstein’s anomaly underwent a cardiovascular magnetic resonance (CMR) due to increased dyspnoea and palpitations. The echocardiogram showed redundant and dysplastic tricuspid leaflets with torrential tricuspid regurgitation (TR; *Figure 1 Panel A*). Vena contracta width was 15 mm, and regurgitant volume and systolic flow reversal in the hepatic vein flow was detected, but the real degree of the regurgitation may not have been fully evaluated. A detailed evaluation of the tricuspid valve was needed as well as a precise assessment of the right ventricle (RV) function; therefore, the patient underwent CMR. During the scan, a noteworthy TR vortex encompassing the right atrium was detected which was better characterized in the additional 4D flow sequences (see Supplementary data online, *Video S1*).

**Figure 1.**
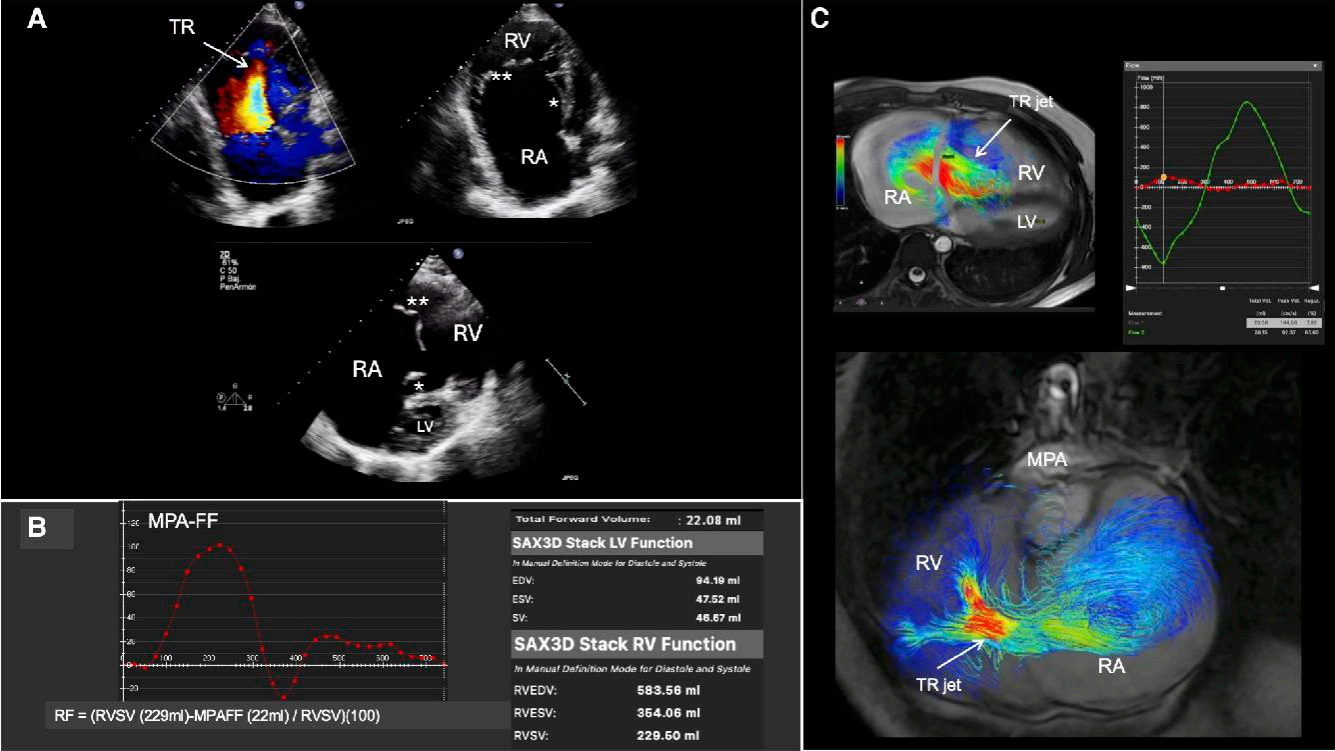
Panel *A*: 2D echocardiogram, RV focused images showing severe tricuspid regurgitation 8 (TR), short axis below. ** Redundant anterior tricuspid leaflet. *Dysplastic septal leaflet. 9 Panel *B*: Main pulmonary artery 2D phase contrast forward flow and curve analysis. LV and 10 RV volumes are showed. 11 Panel *C*: 4D Flow images. 4 chamber view showing the torrential TR jet with a massive 12 vortex occupying the right atrium. Direct TR flow measurement at this level shows 85.6% TR 13 fraction. Torrential TR 3 chamber view.

The TR fraction, determined by subtracting the main pulmonary artery forward flow (using 2D phase contrast images) from the RV stroke volume (*Figure 1 Panel B*), was found to be comparable with the flow directly measured perpendicular to the maximum velocity jet using 4D flow (90% vs. 86%; *Figure 1 Panel C*). Moreover, the measured regurgitant volume confirms that, contrary to previous assumptions, the functional RV is not a small cavity even in Carpentier B Ebstein’s anomaly, rendering end-diastolic volumes up to eight times larger than those of the left ventricle. Another important feature of this image is the clear representation of how CMR enables accurate measurement of ventricular volumes, ejection fraction, and regurgitant volumes, which are essential for assessing the severity of TR and RV dysfunction, with additional assessment of vorticity and haemodynamic parameters.

## Supplementary data

Supplementary data are available at *European Heart Journal - Imaging Methods and Practice* online.

**Data availability:** No new data were generated or analysed in support of this research.

## Lead Author Biography


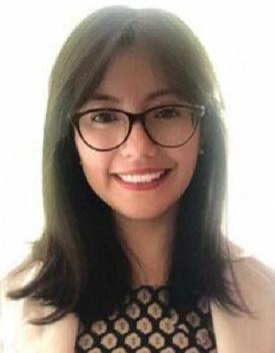


Laura Victoria Torres-Araujo is a young cardiologist working at the National Heart Institute ‘Ignacio Chávez’ in Mexico. Additionally, she serves as a CMR and CT consultant at CT Scanner Lomas Altas. Following the completion of her CMR fellowship at Royal Brompton Hospital, she is currently engaged in imaging and research endeavours within the CMR and CT field.

## Supplementary Material

qyae066_Supplementary_Data

